# FDA approval of Amivantamab-Vmjw in combination with carboplatin and pemetrexed for EGFR-mutated NSCLC: a new therapeutic milestone

**DOI:** 10.1097/MS9.0000000000003003

**Published:** 2025-02-07

**Authors:** Ibrahim Nagmeldin Hassan, Muhsin Nagmeldin Hassan Ibrahim

**Affiliations:** a Faculty of Medicine, University of Khartoum, Khartoum, Sudan; b Faculty of medicine, Sudan University of Science and Technology, Sudan

## Background

Lung cancer remains one of the leading causes of cancer-related mortality worldwide, with non-small cell lung cancer (NSCLC) accounting for approximately 85% of all lung cancer cases. Within this subset, mutations in the epidermal growth factor receptor (EGFR) gene, including exon 19 deletions and exon 21 L858R substitution mutations, are common, driving tumor growth and progression^[^1^]^. These mutations are found in around 10-15% of Caucasian patients and up to 40% of Asian patients with NSCLC, making them key targets for therapy^[^2^]^. Despite advances in targeted treatments, many patients eventually develop resistance, underscoring the need for more effective therapeutic options^[^3^]^.

## Treatment options for NSCLC

Standard treatment options for NSCLC depend on the stage of the disease and the presence of specific genetic mutations^[^4^]^. Targeted therapies, particularly EGFR tyrosine kinase inhibitors (TKIs) like osimertinib, have revolutionized treatment for patients with EGFR-mutant NSCLC^[^5^]^. However, despite initial success, resistance to TKIs inevitably develops, leading to disease progression^[^3^]^. When this occurs, patients often face limited treatment options, typically turning to chemotherapy regimens such as carboplatin and pemetrexed, which offer only modest benefits. As a result, there is a continuous search for more effective treatments that can prolong survival and enhance quality of life.

## Amivantamab-vmjw in combination

The FDA’s recent approval of amivantamab-vmjw in combination with carboplatin and pemetrexed for patients with locally advanced or metastatic NSCLC represents a pivotal advancement in the field^[^6^]^. Amivantamab is a bispecific antibody targeting both EGFR and MET receptors, designed to overcome resistance mechanisms that reduce the effectiveness of earlier EGFR inhibitors like osimertinib^[^7^]^. By combining amivantamab with standard chemotherapy, the treatment targets not only the tumor’s growth signals but also its resistance pathways, offering a comprehensive approach to combat disease progression.

## Comparative analysis and emerging therapies

Amivantamab-vmjw is part of a growing arsenal of treatments for EGFR-mutant NSCLC. Other emerging therapies include mobocertinib and lazertinib, which target EGFR resistance mutations like exon 20 insertions^[^8,9^]^. While direct comparative trials are still underway, early data suggest that amivantamab’s dual targeting of EGFR and MET may provide a broader mechanism of action compared to single-target inhibitors. The MARIPOSA-1 trial is expected to provide further insights into how amivantamab fares against other therapies in head-to-head comparisons.

## Clinical trials and efficacy

The approval of amivantamab-vmjw was based on the results of the MARIPOSA-2 trial, a randomized, open-label study that enrolled 657 patients with EGFR-mutant NSCLC who had progressed after osimertinib treatment^[^6^]^. Patients were randomly assigned to receive either amivantamab with carboplatin and pemetrexed (amivantamab + CP), carboplatin and pemetrexed (CP) alone, or amivantamab as part of another combination regimen. The trial’s primary endpoint was progression-free survival (PFS), with overall response rate (ORR) and overall survival (OS) serving as key secondary outcomes.

The results were impressive: patients in the amivantamab + CP group demonstrated a median PFS of 6.3 months compared to 4.2 months in the CP-alone group. This significant improvement was supported by a hazard ratio of 0.48, indicating a 52% reduction in the risk of disease progression or death. Additionally, the ORR was notably higher in the amivantamab + CP arm (53%) compared to the CP-alone arm (29%), confirming the clinical benefit of adding amivantamab to the treatment regimen. Although the OS data at the second interim analysis did not show a statistically significant difference, the hazard ratio of 0.73 suggested a positive trend that warrants further investigation.

## Safety and side effects

As with any new treatment, safety is a critical consideration. The most common adverse effects of the amivantamab combination therapy included rash, infusion-related reactions, fatigue, gastrointestinal disturbances (including constipation and vomiting), musculoskeletal pain, and COVID-19 infections^[^10^]^. These side effects are consistent with those seen in other EGFR-targeting therapies. While they are generally manageable, clinicians must remain vigilant in monitoring patients and addressing these reactions promptly to ensure the therapy’s benefits outweigh its risks.

Additionally, the risk of serious adverse events such as infusion reactions highlights the importance of premedicating patients with antihistamines and corticosteroids to minimize complications. Close adherence to dosing and administration guidelines is essential^[^7^]^.

## Dosage and precautions

The recommended dosage of amivantamab is weight-based: 1050 mg for patients weighing less than 80 kg and 1400 mg for those weighing 80 kg or more. The drug is administered intravenously weekly for the first 4 weeks, followed by every 2 weeks. Premedication with antihistamines and acetaminophen is necessary to prevent infusion-related reactions. Dose adjustments are advised in cases of severe adverse events or prolonged toxicity.

## Further studies and recommendations

Future research should focus on head-to-head comparisons between amivantamab and other EGFR-targeted therapies. Additionally, studies exploring its role in combination with immune checkpoint inhibitors or as part of sequential treatment strategies may help optimize care. Long-term safety data and real-world effectiveness studies are also needed to confirm its benefits beyond clinical trials.

## Conclusion

The FDA approval of amivantamab-vmjw in combination with carboplatin and pemetrexed offers a new and much-needed option for patients with EGFR-mutant NSCLC who have progressed after prior TKI therapy. The results of the MARIPOSA-2 trial highlight its potential to significantly improve progression-free survival and response rates, providing hope for those with limited treatment options. While the safety profile is consistent with other EGFR-targeted therapies, the benefits of this combination therapy are clear in extending disease control. This approval represents an important step forward in addressing the unmet needs of patients with advanced NSCLC, further solidifying the role of precision medicine in oncology.

## Data Availability

The dataset used and/or analysed during the study is available from the corresponding author upon reasonable request.
